# Next-Generation Genome Sequencing of *Sedum plumbizincicola* Sheds Light on the Structural Evolution of Plastid rRNA Operon and Phylogenetic Implications within Saxifragales

**DOI:** 10.3390/plants8100386

**Published:** 2019-09-29

**Authors:** Hengwu Ding, Ran Zhu, Jinxiu Dong, De Bi, Lan Jiang, Juhua Zeng, Qingyu Huang, Huan Liu, Wenzhong Xu, Longhua Wu, Xianzhao Kan

**Affiliations:** 1 The Institute of Bioinformatics, College of Life Sciences, Anhui Normal University, Wuhu 241000, Anhui, China; hengwuding@ahnu.edu.cn (H.D.); zhuran0911@ahnu.edu.cn (R.Z.); jinxiudong@ahnu.edu.cn (J.D.); jianglan@ahnu.edu.cn (L.J.); j.h.zeng@ahnu.edu.cn (J.Z.); yunshancike@ahnu.edu.cn (Q.H.); 2 The Provincial Key Laboratory of the Conservation and Exploitation Research of Biological Resources in Anhui, Wuhu 241000, Anhui, China; 3 National Engineering Laboratory of Soil Pollution Control and Remediation Technologies, Institute of Soil Science, Chinese Academy of Sciences, Nanjing 210008, Jiangsu, China; wzy@issas.ac.cn (D.B.); 2016203030@njau.edu.cn (H.L.); 4 Key Laboratory of Plant Resources, Institute of Botany, Chinese Academy of Sciences, Beijing 100093, China; xuwzh@ibcas.ac.cn

**Keywords:** *Sedum plumbizincicola*, Crassulaceae, Saxifragales, *rrn* operon, phylogeny

## Abstract

The genus *Sedum*, with about 470 recognized species, is classified in the family Crassulaceae of the order Saxifragales. Phylogenetic relationships within the Saxifragales are still unresolved and controversial. In this study, the plastome of *S. plumbizincicola* was firstly presented, with a focus on the structural analysis of *rrn* operon and phylogenetic implications within the order Saxifragaceae. The assembled complete plastome of *S. plumbizincicola* is 149,397 bp in size, with a typical circular, double-stranded, and quadripartite structure of angiosperms. It contains 133 genes, including 85 protein-coding genes (PCGs), 36 tRNA genes, 8 rRNA genes, and four pseudogenes (one *ycf1*, one *rps19*, and two *ycf15*). The predicted secondary structure of *S. plumbizincicola* 16S rRNA includes three main domains organized in 74 helices. Further, our results confirm that 4.5S rRNA of higher plants is associated with fragmentation of 23S rRNA progenitor. Notably, we also found the sequence of putative *rrn5* promoter has some evolutionary implications within the order Saxifragales. Moreover, our phylogenetic analyses suggested that *S. plumbizincicola* had a closer relationship with *S. sarmentosum* than *S. oryzifolium*, and supported the taxonomic revision of *Phedimus*. Our findings of the present study will be useful for further investigation of the evolution of plastid rRNA operon and phylogenetic relationships within Saxifragales.

## 1. Introduction

The genus *Sedum* comprises more than 420 recognized species, which is the most species-rich member of the family Crassulaceae [1,2]. Some species, formerly classified as *Sedum*, are now assigned to the segregate genera *Hylotelephium* and *Rhodiola* [3,4,5,6]. The family Crassulaceae, together with 14 other family members, has been classified in the order Saxifragales. Recently, increasing research efforts have been focused on the evolution of Saxifragales, however, phylogenetic relationships within the order are still unresolved due apparently to a rapid, ancient radiation [7,8,9,10,11,12].

Extensive genes were transferred from plastids to the nucleus during evolution. In most land plants, the plastid genome (plastome) is a circular biological macromolecule with a typical quadripartite structure [13,14,15]. In higher plants, compared with hundreds or thousands of tandem repeats in nuclear ribosomal RNA genes [16,17,18], typical plastid rRNA genes are characterized by a pair of inverted *rrn* operons, which show the gene order of *rrn16*, *rrn23*, *rrn4.5*, and *rrn5* [19]. With the rapid development of next generation genome sequencing, more and more complete plastid genomes (plastomes) have been deposited in a public database. Recently, a total of over 3000 reference sequences of plastomes were available in GenBank. The plastomes have been widely accepted as a popular tool for phylogenetic studies [7,20,21,22,23,24,25,26].

Thus far, 43 plastomes have been obtained in Saxifragales, as shown in Table 1. Currently, there is no report of a plastome for *S. plumbizincicola*, a well-known Zn/Cd hyperaccumulator, which was newly discovered from lead and zinc mining areas in Zhejiang province, China [27]. In this paper, we sequenced the plastome of this species using next-generation genome sequencing. Together with the public sequences, we performed a comparative analysis of plastomes within Saxifragales. Consequently, the aims of this research were (1) to investigate general features of the *S. plumbizincicola* plastome, (2) to examine the structural evolution of the plastid ribosomal RNA operon, and (3) to clarify phylogenetic relationships within the order Saxifragales.

## 2. Materials and Methods

### 2.1. Sample Collection and DNA Extraction

The fresh leaf samples of *S. plumbizincicola* (code AHNU-KPBK001) were collected from Panjiacun (29°35′16″ N, 118°35′19″ E) in Zhejiang Province, east China. Genomic DNA extraction was conducted using the Plant Genomic DNA kit (Tiangen, Beijing, China), following the manufacturer's instructions. The library was constructed using a TruSeq DNA PCR-Free Library Prep Kit (Illumina, San Diego, CA, USA) and sequenced on the Illumina Hiseq X Ten (Illumina, San Diego, CA, USA) with the strategy of 150 paired-ends and an insert size of 350 bp.

### 2.2. Genome Assembly, Gene Annotation, and Sequence Analyses

The paired-end reads were first checked with Fastqc [28] and then trimmed for quality using Trimmomatic 0.39 [29]. After that, obtained clean reads were filtered and assembled with GetOrganelle 1.5.2 [30] using the chloroplast genome of *S. sarmentosum* [7] as reference. The chloroplast genome was annotated with GeSeq [31]. The secondary cloverleaf structures of tRNAs were identified using tRNA-scan SE web server [32]. The secondary structures of rRNAs were predicted by comparison with those of other plant species [33].

### 2.3. Phylogenetic Analysis

To resolve the phylogenetic relationships among Saxifragales species, two phylogenetic approaches were applied: the maximum likelihood (ML) method in RAxML GUI 1.5b2 [34], as well as the Bayesian inference (BI) method in MrBayes 3.2.7a [35]. With exclusion of the termination codons, 79 protein-coding genes (PCGs) and 4 rRNAs of 37 Saxifragales species were used to construct an evolutionary tree. A phylogenomic study by Yang et al. [36] revealed a sister group relationship between Saxifragales and Rosids. We selected therefore two Vitales species within Rosids (*Vitis heyneana*, NC_039796; *V. vinifera*, NC_007957) as outgroups.

For ML analyses, we performed analyses with thorough bootstrap for ten runs and 1000 replicates under the GTRCAT model using RAxML GUI. For BI analyses, the best-fit models for 83 genes were first selected based on Bayesian information criterion (BIC) values in ModelGenerator 0.85 [53], then two simultaneous runs with eight independent Markov chains were run for 10,000,000 generations (sampling every 1000 generations).

## 3. Results and Discussion

### 3.1. General Features of S. plumbizincicola Plastome

**Figure 1:**
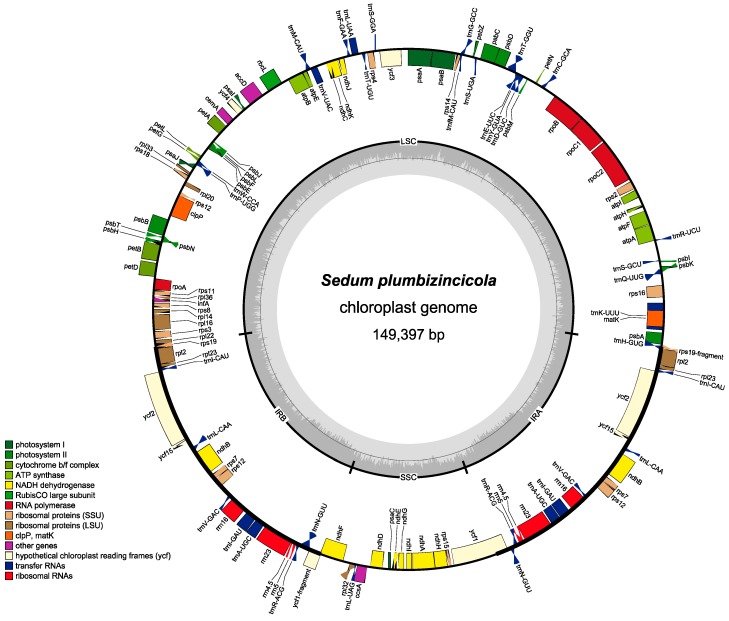
Chloroplast genome annotation map for *Sedum plumbizincicola*. Genes lying outside the circle are transcribed in a clockwise direction, whereas genes inside are transcribed in a counterclockwise direction. Different colors represent different functional groups. The dashed darker and lighter gray in the inner circle denote G + C and A + T contents of chloroplast genome, respectively. LSC, SSC, and IRs mean long single copy, small single copy, and inverted repeat regions, respectively.

Based on Bowtie2 mapping, in total 19,610,999 reads (21.5% of total reads) were mapped to the reference genome (*S. sarmentosum*, NC_023085), with a 1969× mean coverage (min, 1286×, max, 3664×, standard deviation, 71). The assembled complete plastome of *S. plumbizincicola* (accession number: MN185459.1) is 149,397 bp in size, with a typical circular, double-stranded, and quadripartite structure of angiosperms. The plastome has two identical inverted repeats (IRs, 25,565 bp) separated by a small single copy (SSC, 16,669 bp) and a large single copy (LSC, 81,598 bp), as shown in Figure 1. Approximately 52.0%, 4.3%, and 1.83% of the genome encodes for proteins, rRNAs, and tRNAs, respectively. Whereas, the remaining 41.87% are non-coding regions, including introns, intergenic spaces, and pseudogenes.

Along with new data from this study, we comparatively investigated the structures and properties of plastomes from 44 species, representing 11 families in Saxifragales, as shown in Table 1. The size of plastomes of Saxifragales ranges from 147,048 bp (*Phedimus kamtschaticus*) to 160,410 bp (*Liquidambar formosana*), as shown in Table S1, and the total of G + C content varies from 36.40% (*Myriophyllum spicatum*) to 38.55% (*Paeonia brownii*).

The plastome of *S. plumbizincicola* contains 133 genes, including 85 protein-coding genes (PCGs), 36 tRNA genes, 8 rRNA genes, and four pseudogenes (one *ycf1*, one *rps19*, and two *ycf15*). Dong et al. [8] reported that *infA* and *rpl32* have been lost from three species of *Paeonia* plastome (*Paeonia brownii*, *P. suffruticosa*, and *P. obovata*). In this study, comparative analysis showed that these two gene loss events occurred in all eleven plastomes of Paeoniaceae. A possible explanation is that the two functional genes have been transferred to the nucleus [8,47,54,55,56,57,58,59,60]. Furthermore, Dong et al. [8] observed that the intron of *rpl2* was completely lost in *Saxifraga stolonifera*. There are currently about 640 species in 33 genera recognized within the family Saxifragaceae [61]. Interestingly, in the current study, the intron of *rpl2* was detected in all families in Saxifragales, except for 13 species from the examined 8 genera representing the major lineages of Saxifragaceae, as shown in Table S1, which indicates an early loss of this intron within this lineage. Besides Saxifragaceae, nine other independent losses of *rpl2* intron were reported in dicotyledons [62,63,64,65,66,67,68,69,70,71,72,73,74,75]. The two most probable mechanisms of loss of the *rpl2* intron are homologous recombination and gene conversion [64,76,77].

### 3.2. Structure Analyses of Plastid Ribosomal RNA Operon

#### 3.2.1. Structure of 16S rRNA

Similar to most other plants, the size of *S. plumbizincicola rrn16* is 1490 bp. In all Saxifragales species examined, the sizes of *rrn16s* are the same as that of *S. plumbizincicola*, except for the family Paeoniaceae, with an insertion (U) between positions 576 and 577 nts. As shown in Table S2, the G + C content of the *rrn16s* of Saxifragales ranges from 56.5% (*Rhodiola rosea*) to 56.9% (*Fortunearia sinensis*, and *Sinowilsonia henryi*). The average G + C content for typical land plants is 56%, whereas this value falls from 52% to 28% for holoparasitic angiosperms, with an increasingly greater number of mutations [78].

**Figure 2:**
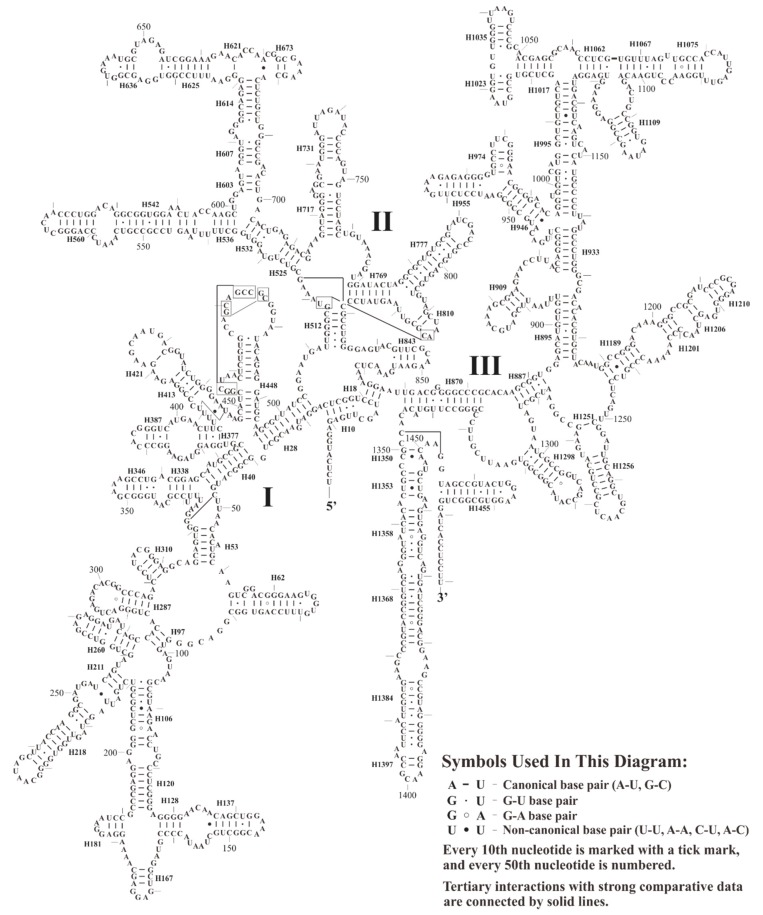
The predicted secondary structure model of 16S rRNA of *S. plumbizincicola*. Roman numbers refer to domain names.

We next examined the predicted secondary structure of 16S rRNA in *S. plumbizincicola*. The structure is similar to the models proposed for other plants [78,79,80], including three main domains organized in 74 helices. In total, 72 mismatched pairs have been detected, and most of them (58/72) are G-U wobble pairs, as shown in Figure 2. Furthermore, we also detected that the position 123 nt of 16S rRNA is cytosine (123-C), whereas other Saxifragales species examined are uracil. To avoid a potential sequencing error, we confirmed the mutation *U123C* by transcriptomic data of *S. plumbizincicola* (accession number: SRR5118122-SRR5118124). For further analysis, the 16S rRNAs from 3125 reference plastomes of land plants deposited in GenBank were investigated. The survey results indicated that only 13 species had the special 123-C, including two hyperaccumulator plants, *Alpinia oxyphylla* and *Curcuma longa* [81,82]. In contrast with non-canonical base pairing (G-U), we particularly observed that the mutation *U123C* of 16S rRNA can form stabilized base pairing (C-G) in helices H120, as shown in Figure 2. However, the underlying biological mechanisms of the mutation *U123C* of 16S rRNA are still unknown.

#### 3.2.2. Structure of 23S rRNA and 4.5S rRNA

As can be seen from Table S2, the size of *rrn23* spans from 2089 bp (*Sedum*) to 2857 bp (*Paeonia suffruticosa*), and the G + C content ranges from 55.0 (*Corylopsis coreana, Loropetalum subcordatum*, and *Chrysosplenium aureobracteatum*) to 55.4% (*M. spicatum*), with an average value of 55.1%. In contrast to *rrn23*, the *rrn4.5* of Saxifragales is remarkably conserved in size (103 bp), with a mean G + C content of 56.7%. The *rrn4.5* and *rrn23* genes are separated by 98–99 bp intergenic spacers (IGd), with G + C content between 57.1% and 60.2%, as shown in Table S2.

**Figure 3:**
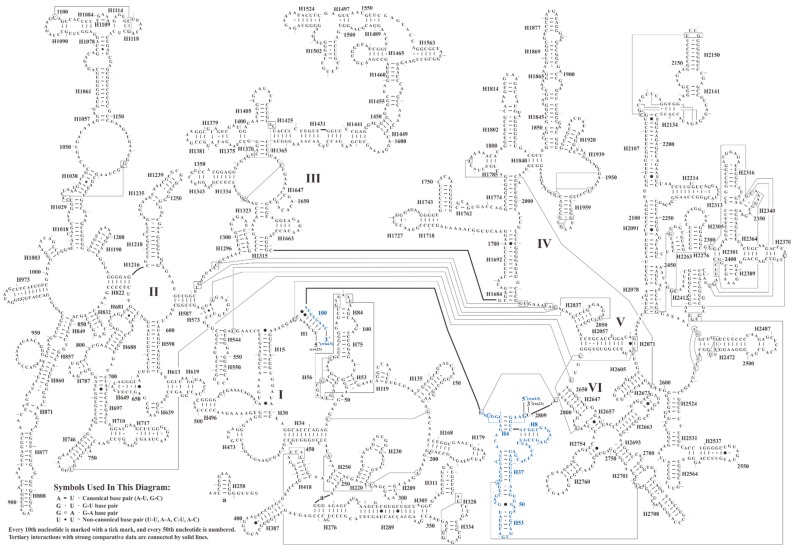
The predicted secondary structure model of 4.5S rRNA and 23S rRNA of *S. plumbizincicola*. Roman numbers refer to domain names. The bases of 4.5S rRNA are presented with blue and the bases of 23S rRNA are presented with black.

**Figure 4:**
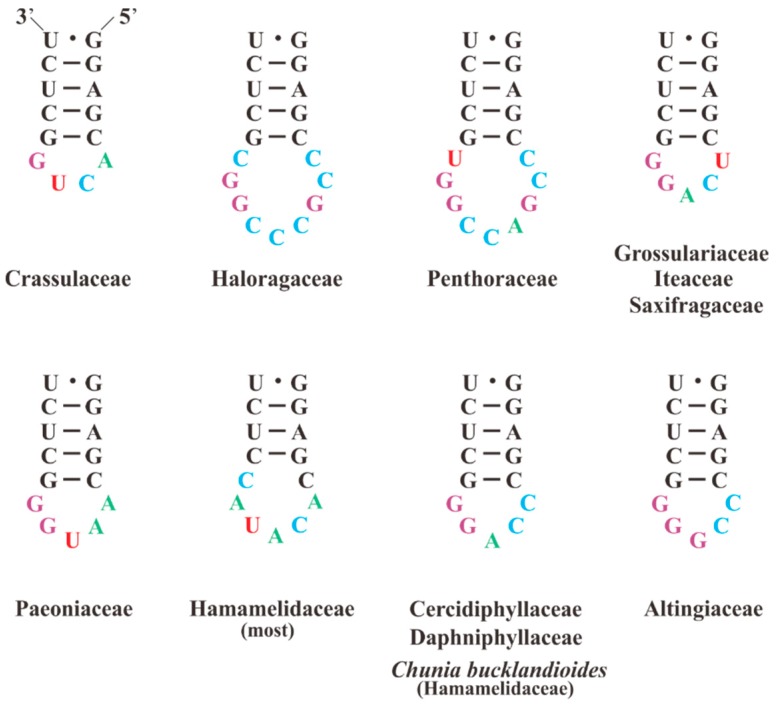
The predicted secondary structure models of H550 of 23S rRNA among Saxifragales species.

**Figure 5:**
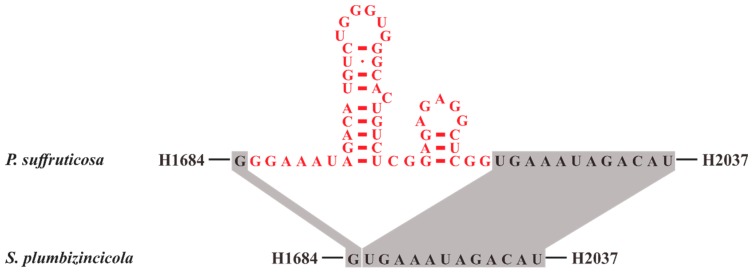
The predicted secondary structure models of the extra insertion between the helices H1684 and H2037 within *Paeonia suffruticosa* 23S rRNA compared with *S. plumbizincicola*. The color red indicates that these bases belong to the extra insertion of *P. suffruticosa*.

**Figure 6:**
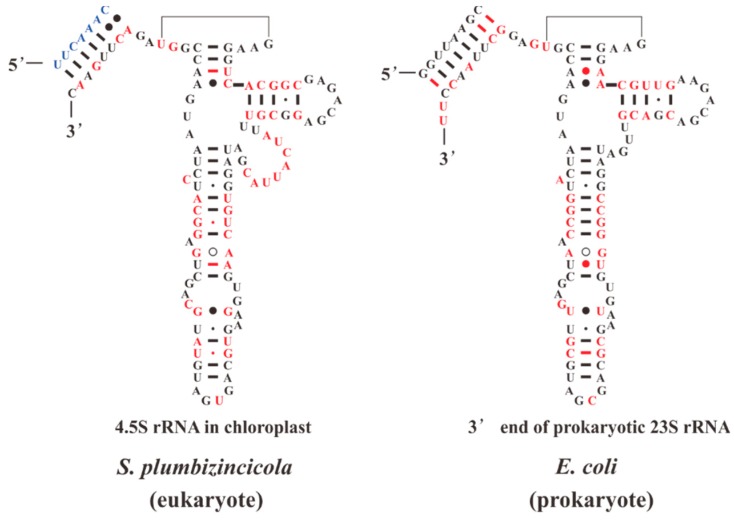
The secondary structure models of the 4.5S rRNA of *S. plumbizincicola* and 3’ end of *Escherichia coli* 23S rRNA. The variations between structures are presented with red. The color blue indicates the bases belonging to 23S rRNA of *S. plumbizincicola*.

The predicted secondary structure of 23S rRNA in *S. plumbizincicola* is similar to the models of Gutell [80,83], containing 149 helices and six domains, as shown in Figure 3. Moreover, a total of 135 mismatched pairs with 101 G-U wobble pairs were found in the structure. We then comparatively analyzed 23S rRNA secondary structures of all investigated taxa in Saxifragales. Remarkably, as shown in Figure 4, the hairpin loops near helix H550 were more divergent than others, including nucleotide substitutions and indels. In particular, these divergent hairpin loops may have potential phylogenetic implications. For instance, all species of Crassulaceae are characterized by six nucleotides (5’-CACUGG-3’) in these hairpin loops. In addition, in contrast to *S. plumbizincicola*, *P. suffruticosa* had an extra 46 nts insertion between the helices H1684 and H2037 of 23S rRNA. Our study further shows that the extra insertion may form two additional helices, as shown in Figure 5. Notably, 4.5S rRNA is a unique component of plastid ribosomes from nonvascular (bryophytes) to vascular plants (pteridophytes, gymnosperms, and angiosperms), which is located on the large subunit. Several previous studies of 4.5S rRNA have failed to find known homologues in other types of ribosomes [84,85,86]. In ongoing follow-up research, 4.5S rRNA has been identified as structurally homologous to the 3’ terminus of bacterial, cyanobacterial, and green algal 23S rRNA [19,84,87,88,89,90]. Based on sequence identity analysis, 4.5S rRNA of *S. plumbizincicola* and 3’ terminus of *Escherichia coli* 23S rRNA (accession number: J01695) share 62.9% nucleotide identity. Interestingly, despite a considerable amount of nucleotide substitutions and indels between these two regions, their secondary structures exhibited similar topology, as shown in Figure 6. This finding confirms once again that 4.5S rRNA of higher plants is associated with fragmentation of 23S rRNA progenitor. 

#### 3.2.3. Structure of 5S rRNA and Evolutionary Implications of Its Putative Promoter

**Figure 7:**
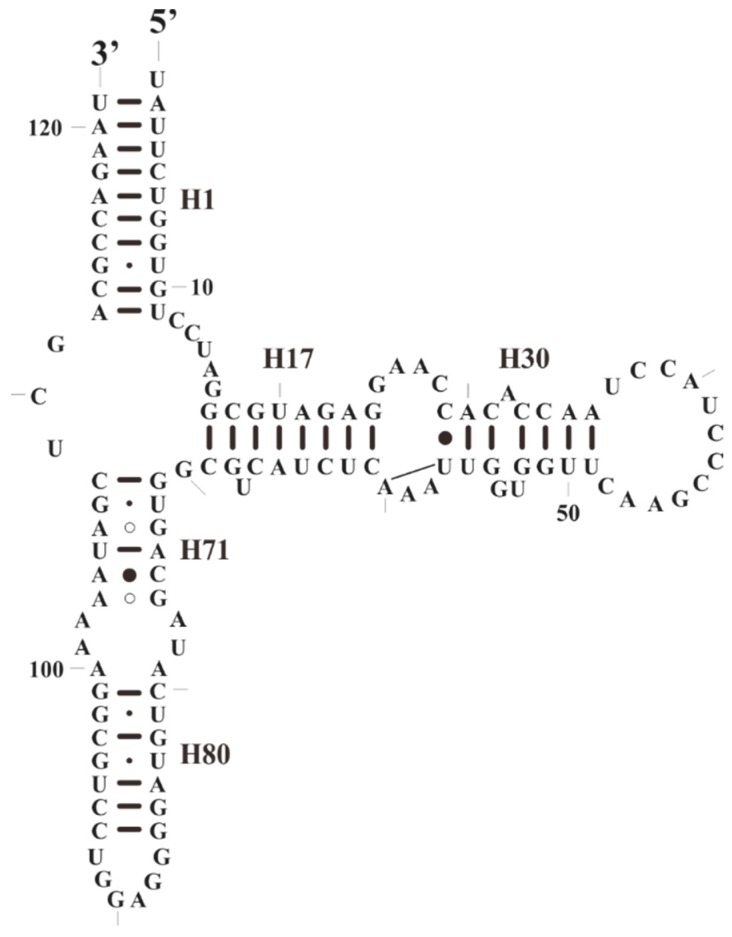
The predicted secondary structure model of 5S rRNA of *S. plumbizincicola*.

Structurally, 5S rRNA is the smallest RNA component of the large ribosomal subunit in all known organisms [91]. In the *S. plumbizincicola* plastome, *rrn5* and *rrn4.5* are physically linked by the intergenic region (IGe), with the size 219 bp, as shown in Table S2. Besides, the predicted secondary structure of *S. plumbizincicola* 5S rRNA is similar to that of other published studies [92,93], harboring five helices, as shown in Figure 7. Furthermore, our comparative sequence analysis identified a perfectly conserved 121-bp *rrn5* among Saxifragales, with medium G + C content (about 52%), as shown in Table S2. In this study, we also used the 5SRNAdb (http://combio.pl/rrna/) to survey the G + C content of plastomic *rrn5*. A total of 839 sequences were downloaded and analyzed. The mean G + C content is 50.73%, with the lowest in *Euglena viridis* (32.26%) and the highest in *Staurastrum punctulatum* (59.84%). The survey shows that there is a great variability in G + C content of *rrn5* for photosynthetic euglenoid and green algae.

**Figure 8:**
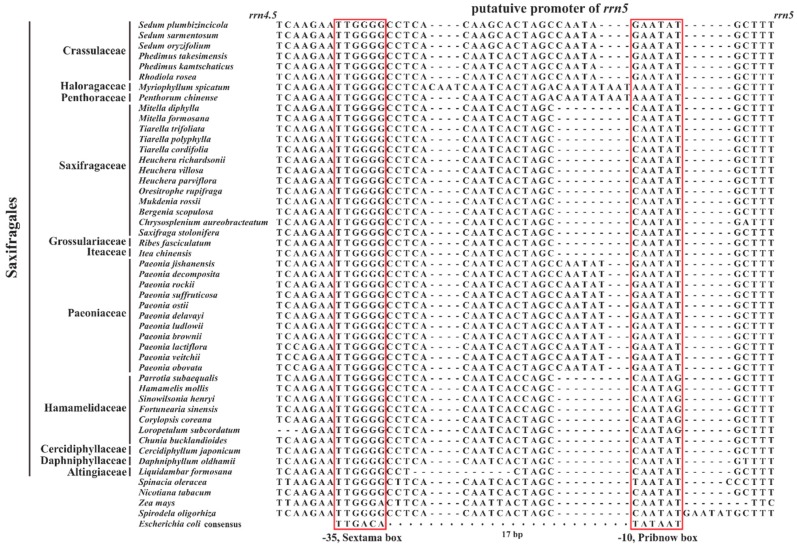
The putative promoters of *rrn5* among Saxifragales species.

Based on similarity of nucleotide sequences, Audren et al. [94] found that a prokaryotic type promoter, which is closely related to the bacterial consensus, was located upstream of the *rrn5* and downstream of the stem-loop structure from spinach. However, the putative promoter is inactive both in vivo and in vitro, likely due to the high GC content of the sextama box (TTGGGG) [94,95]. A number of studies have demonstrated that the 5S rRNA gene is transcribed with the other ribosomal genes within the same operon [19,94,96,97]. Notably, the spinach putative promoter was also detected in the similar region from all 44 Saxifragales species. As shown in Figure 8, it contains a sextama box (−35 region, T_100_T_100_G_100_G_100_G_100_G_100_) and a pribnow box (−10 region, C_57_A_100_A_100_T_100_A_100_T_86_) separated by 8–29 bp within Saxifragales, as shown in Figure 8. Interestingly, we found the sequence of putative *rrn5* promoters have some evolutionary implications. For example, all spacers between -35 and -10 boxes from 44 investigated species share the 16 common nucleotides (CCTCACAATCACTAGC), except for *Liquidambar formosana* (CCTCTAGC). Due to nucleotide insertion, deletion, and substitution, the ancestral sequence was then further evolved to different apomorphies in diversified lineages within Saxifragales.

### 3.3. Phylogenetic Implications

**Figure 9:**
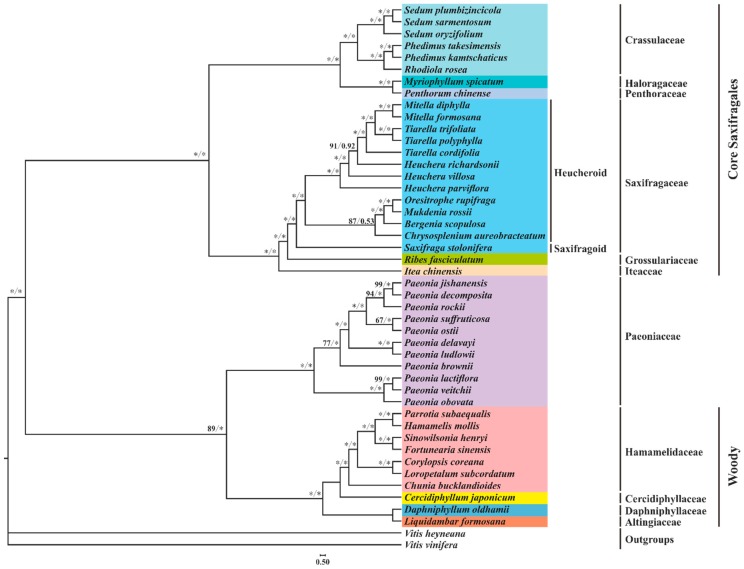
Nucleotide-based phylogenetic tree of 44 Saxifragales species. This analysis is based on 79 protein-coding genes (PCGs) and 4 rRNAs. The maximum likelihood bootstrap (BS) and bayesian posterior probability (PP) values for each node are indicated; * indicates 100% bootstrap or 1.00 PP. The bootstrap percentages < 50% and posterior probabilities < 0.5 were omitted.

To investigate the evolutionary relationships among the order Saxifragales, we performed phylogenetic analyses using 83 plastid genes of 44 species. Two species of Vitaceae (*V. heyneana* and *V. vinifera*) were employed as outgroups. After alignment, the concatenated sequences are 74,751 bp long. The trees derived from ML and BI analyses display the same topology, as shown in Figure 9. According to the Angiosperm Phylogeny Group (APG) system IV [98], the order Saxifragales comprises 15 families, 11 of which were chosen for the phylogenetic analyses. The order Saxifragales can be generally divided into two clades: core Saxifragales clade (maximum likelihood bootstrap [BS] = 100 and bayesian posterior probability [PP] = 1.0) and Paeoniaceae plus the woody clade ([BS] = 89 and [PP] = 1.0). The former clade is subdivided into two subclades: one containing Crassulaceae, Haloragaceae, and Penthoraceae, and the other comprising three families of Saxifragaceae alliance (Grossulariaceae, Saxifragaceae, and Iteaceae). The latter clade includes Paeoniaceae, Altingiaceae, Cercidiphyllaceae, Daphniphyllaceae, and Hamamelidaceae. In general, the framework of relationships within Saxifragales generated from this study agrees with those reported by Jian et al. [11], Moore et al. [99], and Soltis et al. [12].

In the present study, we found that *S. plumbizincicola* had a closer relationship with *S. sarmentosum* than *S. oryzifolium*. Furthermore, *Sedum* is sister to (*Phedimus* + *Rhodiola*). Species of *Phedimus*, previously treated as members of *Sedum*, have been classified as a separate genus [100,101]. Our data support this taxonomic revision of *Phedimus*.

Within Saxifragaceae alliance, Iteaceae is sister to (Grossulariaceae + Saxifragaceae), with strongly supported nodes ([BS] = 100 and [PP] = 1.0). Furtherly, Saxifragaceae can be divided into two subclades: heucheroid and saxifragoid [61,101]. Within the heucheroid, two genera, *Heuchera* and *Tiarella*, have been suggested as polyphyletic by several chloroplast markers [102,103]. Our present study based on nearly whole plastome sequence data supported this view. However, both morphology and nuclear internal transcribed spacers (ITS) data have indicated that the two genera are monophyletic [102,104,105]. This incongruence between chloroplast and nuclear gene trees may be due to chloroplast capture [105,106,107,108,109,110].

Our results also accepted the monophyly of the woody clade, which is sister to the family Paeoniaceae. It is noteworthy that deep-level relationships within Hamamelidaceae are strongly supported. Nevertheless, the closest relatives of this family and relationships among these woody families are still unresolved in our analysis. This might partially be attributed to an ancient, rapid radiation [11]. Therefore, further detailed analyses need be conducted to evaluate the relationships within the woody clade.

## 4. Conclusions

In the present study, we first sequenced and analyzed the plastome of *S. plumbizincicola*. The genome structure and gene order were revealed, including 85 PCGs, 36 tRNA genes, 8 rRNA genes, and four pseudogenes. Next, we focused on the analyses of the primary and secondary structures of plastid rRNA genes. Notably, we found the sequence of putative *rrn5* promoter has some evolutionary implications within the order Saxifragales. Based on the 83 plastid genes from 44 species, phylogenetic analyses demonstrated that *S. plumbizincicola* had a closer relationship with *S. sarmentosum* than *S. oryzifolium*. Our findings reported here shed light on the structural evolution of plastid rRNA operon and phylogenetic relationships within Saxifragales.

## Figures and Tables

**Table 1:** Species of plastomes examined in this study.

Family	Species	Accession No.	Reference
Altingiaceae	*Liquidambar formosana*	NC_023092.1	[7]
Cercidiphyllaceae	*Cercidiphyllum japonicum*	NC_037940.1	[37]
Crassulaceae	*Phedimus kamtschaticus*	NC_037946.1	[38]
Crassulaceae	*Phedimus takesimensis*	NC_026065.1	Unpublished
Crassulaceae	*Rhodiola rosea*	NC_041671.1	[39]
Crassulaceae	*Sedum oryzifolium*	NC_027837.1	Unpublished
**Crassulaceae**	***Sedum plumbizincicola***	**MN185459.1**	**This study**
Crassulaceae	*Sedum sarmentosum*	NC_023085.1	[7]
Daphniphyllaceae	*Daphniphyllum oldhamii*	NC_037883.1	[8]
Grossulariaceae	*Ribes fasciculatum*	MH191388.1	[8]
Haloragaceae	*Myriophyllum spicatum*	NC_037885.1	[8]
Hamamelidaceae	*Chunia bucklandioides*	NC_041163.1	[40]
Hamamelidaceae	*Corylopsis coreana*	NC_040141.1	[41]
Hamamelidaceae	*Fortunearia sinensis*	NC_041487.1	[42]
Hamamelidaceae	*Hamamelis mollis*	NC_037881.1	[8]
Hamamelidaceae	*Loropetalum subcordatum*	NC_037694.1	[43]
Hamamelidaceae	*Parrotia subaequalis*	NC_037243.1	Unpublished
Hamamelidaceae	*Sinowilsonia henryi*	NC_036069.1	Unpublished
Paeoniaceae	*Paeonia brownii*	NC_037880.1	[8]
Paeoniaceae	*Paeonia decomposita*	NC_039425.1	[44]
Paeoniaceae	*Paeonia delavayi*	NC_035718.1	[45]
Paeoniaceae	*Paeonia jishanensis*	MG991935.1	[46]
Paeoniaceae	*Paeonia lactiflora*	NC_040983.1	[47]
Paeoniaceae	*Paeonia ludlowii*	NC_035623.1	[45]
Paeoniaceae	*Paeonia obovata*	NC_026076.1	Unpublished
Paeoniaceae	*Paeonia ostii*	NC_036834.1	Unpublished
Paeoniaceae	*Paeonia rockii*	NC_037772.1	[48]
Paeoniaceae	*Paeonia suffruticosa*	NC_037879.1	[8]
Paeoniaceae	*Paeonia veitchii*	NC_032401.1	Unpublished
Penthoraceae	*Penthorum chinense*	NC_023086.1	[7]
Iteaceae	*Itea chinensis*	NC_037884.1	[8]
Saxifragaceae	*Bergenia scopulosa*	NC_036061.1	[49]
Saxifragaceae	*Chrysosplenium aureobracteatum*	NC_039740.1	[50]
Saxifragaceae	*Heuchera parviflora*	KR478645.1	[51]
Saxifragaceae	*Heuchera richardsonii*	NC_042923.1	Unpublished
Saxifragaceae	*Heuchera villosa*	NC_042924.1	Unpublished
Saxifragaceae	*Mitella diphylla*	NC_042925.1	Unpublished
Saxifragaceae	*Mitella formosana*	NC_042926.1	Unpublished
Saxifragaceae	*Mukdenia rossii*	NC_037495.1	Unpublished
Saxifragaceae	*Oresitrophe rupifraga*	NC_037514.1	[52]
Saxifragaceae	*Saxifraga stolonifera*	NC_037882.1	[8]
Saxifragaceae	*Tiarella cordifolia*	NC_042927.1	Unpublished
Saxifragaceae	*Tiarella polyphylla*	NC_042928.1	Unpublished
Saxifragaceae	*Tiarella trifoliata*	NC_042929.1	Unpublished

## Supplementary Materials

The following are available online at https://www.mdpi.com/2223-7747/8/10/386/s1, Table S1: Genomic characteristics of 44 complete Saxifragales plastomes, Table S2: A comparison of sizes, G + C contents in the different regions of ribosomal RNA operon from Saxifragales plastomes.

## References

[1] Nikulin V.Y., Gontcharova S.B., Stephenson R., Gontcharov A.A. (2016). Phylogenetic relationships between *Sedum* L. and related genera (Crassulaceae) based on ITS rDNA sequence comparisons. Flora.

[2] Thiede J., Eggli U., Kubitzki K. (2007). Crassulaceae. The Families and Genera of Vascular Plants.

[3] Zhang J., Meng S., Wen J., Rao G. (2014). Phylogenetic relationships and character evolution of *Rhodiola* (Crassulaceae) based on nuclear ribosomal ITS and plastid trnL-F and psbA-trnH sequences. Syst. Bot..

[4] Berger A., Engler A., Prantl K. (1930). Crassulaceae. Die Natürlichen Pflanzenfamilien.

[5] Borissova A. (1939). Crassulaceae. Flora USSR.

[6] Ohba H. (1978). Generic and infrageneric classification of the Old World Sedoideae (Crassulaceae). J. Fac. Sci. Univ. Tokyo 3.

[7] Dong W., Xu C., Cheng T., Lin K., Zhou S. (2013). Sequencing angiosperm plastid genomes made easy: A complete set of universal primers and a case study on the phylogeny of Saxifragales. Genome Biol. Evol..

[8] Dong W., Xu C., Wu P., Cheng T., Yu J., Zhou S., Hong D.-Y. (2018). Resolving the systematic positions of enigmatic taxa: Manipulating the chloroplast genome data of Saxifragales. Mol. Phylogenet. Evol..

[9] Fishbein M., Hibsch-Jetter C., Soltis D.E., Hufford L. (2001). Phylogeny of Saxifragales (angiosperms, eudicots): Analysis of a rapid, ancient radiation. Syst. Biol..

[10] Fishbein M., Soltis D.E. (2004). Further resolution of the rapid radiation of Saxifragales (angiosperms, eudicots) supported by mixed-model Bayesian analysis. Syst. Bot..

[11] Jian S., Soltis P.S., Gitzendanner M.A., Moore M.J., Li R., Hendry T.A., Qiu Y.-L., Dhingra A., Bell C.D., Soltis D.E. (2008). Resolving an ancient, rapid radiation in Saxifragales. Syst. Biol..

[12] Soltis D.E., Mort M.E., Latvis M., Mavrodiev E.V., O’Meara B.C., Soltis P.S., Burleigh J.G., Rubio de Casas R. (2013). Phylogenetic relationships and character evolution analysis of Saxifragales using a supermatrix approach. Am. J. Bot..

[13] Cui Y., Nie L., Sun W., Xu Z., Wang Y., Yu J., Song J., Yao H. (2019). Comparative and Phylogenetic Analyses of Ginger (*Zingiber officinale*) in the Family Zingiberaceae Based on the Complete Chloroplast Genome. Plants.

[14] Huang Y., Yang Z., Huang S., An W., Li J., Zheng X. (2019). Comprehensive Analysis of *Rhodomyrtus tomentosa* Chloroplast Genome. Plants.

[15] Yang Z., Huang Y., An W., Zheng X., Huang S., Liang L. (2019). Sequencing and Structural Analysis of the Complete Chloroplast Genome of the Medicinal Plant *Lycium chinense* Mill. Plants.

[16] Eickbush T.H., Eickbush D.G. (2007). Finely orchestrated movements: Evolution of the ribosomal RNA genes. Genetics.

[17] Kan X.Z., Wang S.S., Ding X., Wang X.Q. (2007). Structural evolution of nrDNA ITS in Pinaceae and its phylogenetic implications. Mol. Phylogenet. Evol..

[18] Nei M., Rooney A.P. (2005). Concerted and birth-and-death evolution of multigene families. Annu. Rev. Genet..

[19] Delp G., Kössel H., Bogorad L., Vasil I.K. (1991). rRNAs and rRNA genes of plastids. Cell Culture and Somatic Cell Genetics of Plants.

[20] Turmel M., Otis C., Lemieux C. (1999). The complete chloroplast DNA sequence of the green alga *Nephroselmis olivacea*: Insights into the architecture of ancestral chloroplast genomes. Proc. Natl. Acad. Sci. USA.

[21] De Cambiaire J.-C., Otis C., Lemieux C., Turmel M. (2006). The complete chloroplast genome sequence of the chlorophycean green alga *Scenedesmus obliquus* reveals a compact gene organization and a biased distribution of genes on the two DNA strands. BMC Evol. Biol..

[22] Turmel M., Otis C., Lemieux C. (2006). The chloroplast genome sequence of *Chara vulgaris* sheds new light into the closest green algal relatives of land plants. Mol. Biol. Evol..

[23] Pombert J.-F., Otis C., Lemieux C., Turmel M. (2005). The chloroplast genome sequence of the green alga *Pseudendoclonium akinetum* (Ulvophyceae) reveals unusual structural features and new insights into the branching order of chlorophyte lineages. Mol. Biol. Evol..

[24] Yan D., Wang Y., Murakami T., Shen Y., Gong J., Jiang H., Smith D.R., Pombert J.-F., Dai J., Wu Q. (2015). *Auxenochlorella protothecoides* and *Prototheca wickerhamii* plastid genome sequences give insight into the origins of non-photosynthetic algae. Sci. Rep..

[25] Ren T., Yang Y., Zhou T., Liu Z.-L. (2018). Comparative plastid genomes of *primula* species: Sequence divergence and phylogenetic relationships. Int. J. Mol. Sci..

[26] Ren T., Zheng W., Han K., Zeng S., Zhao J., Liu Z.-L. (2017). Characterization of the complete chloroplast genome sequence of *Lysionotus pauciflorus* (Gesneriaceae). Conserv. Genet. Resour..

[27] Wu L., Liu Y., Zhou S., Guo F., Bi D., Guo X., Baker A., Smith J., Luo Y. (2013). *Sedum plumbizincicola* XH Guo et SB Zhou ex LH Wu (Crassulaceae): A new species from Zhejiang Province, China. Plant Syst. Evol..

[28] Andrews S. FastQC: A Quality Control Tool for High Throughput Sequence Data (2010). http://www.bioinformatics.babraham.ac.uk/projects/fastqc/.

[29] Bolger A.M., Lohse M., Usadel B. (2014). Trimmomatic: A flexible trimmer for Illumina sequence data. Bioinformatics.

[30] Jin J.-J., Yu W.-B., Yang J.-B., Song Y., Yi T.-S., Li D.-Z. (2018). GetOrganelle: A simple and fast pipeline for de novo assembly of a complete circular chloroplast genome using genome skimming data. bioRxiv.

[31] Tillich M., Lehwark P., Pellizzer T., Ulbricht-Jones E.S., Fischer A., Bock R., Greiner S. (2017). GeSeq–versatile and accurate annotation of organelle genomes. Nucleic Acids Res..

[32] Lowe T.M., Chan P.P. (2016). tRNAscan-SE On-line: Integrating search and context for analysis of transfer RNA genes. Nucleic Acids Res..

[33] Cannone J.J., Subramanian S., Schnare M.N., Collett J.R., D’Souza L.M., Du Y., Feng B., Lin N., Madabusi L.V., Müller K.M. (2002). The comparative RNA web (CRW) site: An online database of comparative sequence and structure information for ribosomal, intron, and other RNAs. BMC Bioinform..

[34] Silvestro D., Michalak I. (2012). raxmlGUI: A graphical front-end for RAxML. Org. Divers. Evol..

[35] Ronquist F., Teslenko M., Van Der Mark P., Ayres D.L., Darling A., Höhna S., Larget B., Liu L., Suchard M.A., Huelsenbeck J.P. (2012). MrBayes 3.2: Efficient Bayesian phylogenetic inference and model choice across a large model space. Syst. Biol..

[36] Yang X., Hu R., Yin H., Jenkins J., Shu S., Tang H., Liu D., Weighill D.A., Yim W.C., Ha J. (2017). The *Kalanchoë* genome provides insights into convergent evolution and building blocks of crassulacean acid metabolism. Nat. Commun..

[37] Qin H., Duan N., Wang M.-B., Liu B.-B. (2018). Complete chloroplast genome of *Cercidiphyllum japonicum* (Cercidiphyllaceae), a tertiary relic endangered tree. Conserv. Genet. Resour..

[38] Seo H.-S., Kim S.-C. (2018). The complete chloroplast genome sequence of *Phedimus Kamtschaticus* (Crassulaceae) in Korea. Mitochondrial DNA Part B.

[39] Zhao D.-N., Zhang J.-Q. (2018). Characterization of the complete chloroplast genome of the traditional medicinal plants *Rhodiola rosea* (Saxifragales: Crassulaceae). Mitochondrial DNA Part B.

[40] Zhou Q., Chen Y., Dai J., Wang F., Wu W., Fan Q., Zhou R., Ng W.L. (2018). The chloroplast genome of *Chunia bucklandioides* (Hamamelidaceae): A rare tree endemic to Hainan, China. Conserv. Genet. Resour..

[41] Choi K.S., Ha Y.-H., Jeong K.S., Joo M., Chang K.S., Choi K. (2019). The complete chloroplast genome of *Corylopsis coreana* (Hamamelidaceae). Conserv. Genet. Resour..

[42] Xu Y., Xiao T.-W., Zhao N., Li T., Liu T.-J., Yan H.-F. (2019). Characterization of the complete plastid genome of an endangered species *Fortunearia sinensis* (Hamamelidaceae). Mitochondrial DNA Part B.

[43] Zhang Y., Cai H., Dong J., Gong W., Li P., Wang Z. (2018). The complete chloroplast genome of *Loropetalum subcordatum*, a national key protected species in China. Conserv. Genet. Resour..

[44] Chen Y., Zhou Q., Sun L., Ng W.L., Zhou R., Wu W. (2019). The chloroplast genome of *Paeonia decomposita* (Paeoniaceae), an endangered wild tree peony from Sichuan, China. Conserv. Genet. Resour..

[45] Li H., Guo Q., Zheng W. (2018). Characterization of the complete chloroplast genomes of two sister species of *Paeonia*: Genome structure and evolution. Conserv. Genet. Resour..

[46] Zhou X.-J., Song L.-L., Peng Z.-F., Sun S.-S., Ya H.-Y., Cheng Y.-W., Zhang Y.-Z. (2019). The complete chloroplast genome sequence of *Paeonia jishanensis* (Paeoniaceae), a rare wild tree peony. Mitochondrial DNA Part B.

[47] Samigullin T.H., Logacheva M.D., Degtjareva G.V., Efimov S.V., Terentieva E.I., Vallejo-Roman C.M. (2018). Complete plastome sequence of *Paeonia lactiflora* Pall. (Paeoniaceae: Saxifragales). Mitochondrial DNA Part B.

[48] Bai G., Guo H., Zhao N., Li S., Zhang Y. (2018). The complete chloroplast genome of *Paeonia rockii* (Paeoniaceae), an endangered endemic species to China. Conserv. Genet. Resour..

[49] Bai G., Fang L., Li S., Cui X. (2018). Characterization of the complete chloroplast genome sequence of *Bergenia scopulosa* (Saxifragales: Saxifragaceae). Conserv. Genet. Resour..

[50] Kim Y.-I., Lee J.-H., Kim Y.-D. (2018). The complete chloroplast genome of a Korean endemic plant *Chrysosplenium aureobracteatum* YI Kim & YD Kim (Saxifragaceae). Mitochondrial DNA Part B.

[51] Folk R.A., Mandel J.R., Freudenstein J.V. (2015). A protocol for targeted enrichment of intron--containing sequence markers for recent radiations: A phylogenomic example from *Heuchera* (Saxifragaceae). Appl. Plant Sci..

[52] Liu L., Wang Y., He P., Li P., Lee J., Soltis D.E., Fu C. (2018). Chloroplast genome analyses and genomic resource development for epilithic sister genera *Oresitrophe* and *Mukdenia* (Saxifragaceae), using genome skimming data. BMC Genom..

[53] Keane T.M., Creevey C.J., Pentony M.M., Naughton T.J., Mclnerney J.O. (2006). Assessment of methods for amino acid matrix selection and their use on empirical data shows that ad hoc assumptions for choice of matrix are not justified. BMC Evol. Biol..

[54] Millen R.S., Olmstead R.G., Adams K.L., Palmer J.D., Lao N.T., Heggie L., Kavanagh T.A., Hibberd J.M., Gray J.C., Morden C.W. (2001). Many parallel losses of infA from chloroplast DNA during angiosperm evolution with multiple independent transfers to the nucleus. Plant Cell.

[55] Jansen R.K., Ruhlman T.A., Bock R., Knoop V. (2012). Plastid genomes of seed plants. Genomics of Chloroplasts and Mitochondria, Advances in Photosynthesis and Respiration.

[56] Liu J., Qi Z.-C., Zhao Y.-P., Fu C.-X., Xiang Q.-Y.J. (2012). Complete cpDNA genome sequence of *Smilax china* and phylogenetic placement of Liliales-Influences of gene partitions and taxon sampling. Mol. Phylogenet. Evol..

[57] Ueda M., Fujimoto M., Arimura S.-I., Murata J., Tsutsumi N., Kadowaki K.-I. (2007). Loss of the rpl32 gene from the chloroplast genome and subsequent acquisition of a preexisting transit peptide within the nuclear gene in *Populus*. Gene.

[58] Park S., Jansen R.K., Park S. (2015). Complete plastome sequence of *Thalictrum coreanum* (Ranunculaceae) and transfer of the rpl32 gene to the nucleus in the ancestor of the subfamily Thalictroideae. BMC Plant Biol..

[59] Jo S., Kim H.-W., Kim Y.-K., Sohn J.-Y., Cheon S.-H., Kim K.-J. (2017). The complete plastome of tropical fruit *Garcinia mangostana* (Clusiaceae). Mitochondrial DNA Part B.

[60] Dong W., Xu C., Cheng T., Zhou S. (2013). Complete chloroplast genome of *Sedum sarmentosum* and chloroplast genome evolution in Saxifragales. PLoS ONE.

[61] Deng J.-B., Drew B.T., Mavrodiev E.V., Gitzendanner M.A., Soltis P.S., Soltis D.E. (2015). Phylogeny, divergence times, and historical biogeography of the angiosperm family Saxifragaceae. Mol. Phylogenet. Evol..

[62] Downie S.R., Olmstead R.G., Zurawski G., Soltis D.E., Soltis P.S., Watson J.C., Palmer J.D. (1991). Six independent losses of the chloroplast DNA rpl2 intron in dicotyledons: Molecular and phylogenetic implications. Evolution.

[63] Gu C., Ma L., Wu Z., Chen K., Wang Y. (2019). Comparative analyses of chloroplast genomes from 22 Lythraceae species: Inferences for phylogenetic relationships and genome evolution within Myrtales. BMC Plant Biol..

[64] Gu C., Tembrock L.R., Johnson N.G., Simmons M.P., Wu Z. (2016). The complete plastid genome of *Lagerstroemia fauriei* and loss of rpl2 intron from *Lagerstroemia* (Lythraceae). PLoS ONE.

[65] Gu C., Tembrock L.R., Wu Z. (2016). Chloroplast genome sequence of *Lagerstroemia guilinensis* (Lythraceae, Myrtales), a species endemic to the Guilin limestone area in Guangxi Province, China. Genome Announc..

[66] Nevill P.G., Howell K.A., Cross A.T., Williams A.V., Zhong X., Tonti-Filippini J., Boykin L.M., Dixon K.W., Small I. (2019). Plastome-wide rearrangements and gene losses in carnivorous Droseraceae. Genome Biol. Evol..

[67] Njuguna A.W., Li Z.-Z., Saina J.K., Munywoki J.M., Gichira A.W., Gituru R.W., Wang Q.-F., Chen J.-M. (2019). Comparative analyses of the complete chloroplast genomes of *nymphoides* and *menyanthes* species (menyanthaceae). Aquat. Bot..

[68] Rabah S.O., Lee C., Hajrah N.H., Makki R.M., Alharby H.F., Alhebshi A.M., Sabir J.S., Jansen R.K., Ruhlman T.A. (2017). Plastome sequencing of ten nonmodel crop species uncovers a large insertion of mitochondrial DNA in cashew. Plant Genome.

[69] Sloan D.B., Alverson A.J., Wu M., Palmer J.D., Taylor D.R. (2012). Recent acceleration of plastid sequence and structural evolution coincides with extreme mitochondrial divergence in the angiosperm genus *Silene*. Genome Biol. Evol..

[70] Wang Y.-H., Wicke S., Wang H., Jin J.-J., Chen S.-Y., Zhang S.-D., Li D.-Z., Yi T.-S. (2018). Plastid genome evolution in the early-diverging legume subfamily Cercidoideae (Fabaceae). Front. Plant Sci..

[71] Wicke S., Müller K.F., de Pamphilis C.W., Quandt D., Wickett N.J., Zhang Y., Renner S.S., Schneeweiss G.M. (2013). Mechanisms of functional and physical genome reduction in photosynthetic and nonphotosynthetic parasitic plants of the broomrape family. Plant Cell.

[72] Xu C., Dong W., Li W., Lu Y., Xie X., Jin X., Shi J., He K., Suo Z. (2017). Comparative analysis of six *Lagerstroemia* complete chloroplast genomes. Front. Plant Sci..

[73] Xue Z.-Q., Xue J.-H., Victorovna K.M., Ma K.-P. (2017). The complete chloroplast DNA sequence of *Trapa maximowiczii* Korsh. (Trapaceae), and comparative analysis with other Myrtales species. Aquat. Bot..

[74] Zhang J., Ruhlman T.A., Sabir J.S., Blazier J.C., Weng M.-L., Park S., Jansen R.K. (2016). Coevolution between nuclear-encoded DNA replication, recombination, and repair genes and plastid genome complexity. Genome Biol. Evol..

[75] Zhao Y., Lu D., Han R., Wang L., Qin P. (2018). The complete chloroplast genome sequence of the shrubby cinquefoil *Dasiphora fruticosa* (Rosales: Rosaceae). Conserv. Genet. Resour..

[76] Fink G.R. (1987). Pseudogenes in yeast?. Cell.

[77] Dujon B. (1989). Group I introns as mobile genetic elements: Facts and mechanistic speculations—A review. Gene.

[78] Nickrent D.L., Duff R.J., Konings D. (1997). Structural analyses of plastid-derived 16S rRNAs in holoparasitic angiosperms. Plant Mol. Biol..

[79] Gutell R.R. (1994). Collection of small subunit (16S-and 16S-like) ribosomal RNA structures: 1994. Nucleic Acids Res..

[80] Gutell R.R. (1993). Comparative studies of RNA: Inferring higher-order structure from patterns of sequence variation. Curr. Opin. Struct. Biol..

[81] Zeiner M., Cindrić I.J. (2017). Review–trace determination of potentially toxic elements in (medicinal) plant materials. Anal. Methods.

[82] Kumar N., Soni H. (2007). Characterization of heavy metals in vegetables using inductive coupled plasma analyzer (ICPA). J. Appl. Sci. Environ. Manag..

[83] Gutell R.R., Gray M.W., Schnare M.N. (1993). A compilation of large subunit (23S and 23S-like) ribosomal RNA structures: 1993. Nucleic Acids Res..

[84] MacKay R.M. (1981). The origin of plant chloroplast 4.5 S ribosomal RNA. FEBS Lett..

[85] Bowman C.M., Dyer T. (1979). 4.5 S ribonucleic acid, a novel ribosome component in the chloroplasts of flowering plants. Biochem. J..

[86] Whitfeld P.R., Leaver C.J., Bottomley W., Atchison B. (1978). Low-molecular-weight (4.5 S) ribonucleic acid in higher-plant chloroplast ribosomes. Biochem. J..

[87] Kumagai I., Pieler T., Subramanian A.R., Erdmann V.A. (1982). Nucleotide sequence and secondary structure analysis of spinach chloroplast 4.5 S RNA. J. Biol. Chem..

[88] Takaiwa F., Kusuda M., Sugiura M. (1982). The nucleotide sequence of chloroplast 4.5 S rRNA from a fern, *Dryopteris acuminata*. Nucleic Acids Res..

[89] Edwards K., Kdssel H. (1981). The rRNA operon from *Zea mays* chloroplasts: Nucleotide sequence of 23S rDNA and its homology with *E. coli* 23S rDNA. Nucleic Acids Res..

[90] Clark C.G., Gerbi S.A. (1982). Ribosomal RNA evolution by fragmentation of the 23S progenitor: Maturation pathway parallels evolutionary emergence. J. Mol. Evol..

[91] Szymanski M., Zielezinski A., Barciszewski J., Erdmann V.A., Karlowski W.M. (2015). 5SRNAdb: An information resource for 5S ribosomal RNAs. Nucleic Acids Res..

[92] Szymanski M., Barciszewska M.Z., Erdmann V.A., Barciszewski J. (2002). 5S ribosomal RNA database. Nucleic Acids Res..

[93] Delihas N., Andersen J. (1982). Generalized structures of the 5S ribosomal RNAs. Nucleic Acids Res..

[94] Audren H., Bisanz-Seyer C., Briat J.-F., Mache R. (1987). Structure and transcription of the 5S rRNA gene from spinach chloroplasts. Curr. Genet..

[95] Hawley D.K., McClure W.R. (1983). Compilation and analysis of *Escherichia coli* promoter DNA sequences. Nucleic Acids Res..

[96] Strittmauer G., Kössel H. (1984). Cotranscription and processing of 23S, 4.5 S and 5S rRNA in chloroplasts from *Zea mays*. Nucleic Acids Res..

[97] Leal-Klevezas D.S., Martínez-Soriano J.P., Nazar R.N. (2000). Cotranscription of 5S rRNA–tRNA^Arg^ (ACG) from *Brassica napus* chloroplasts and processing of their intergenic spacer. Gene.

[98] Chase M.W., Christenhusz M., Fay M., Byng J., Judd W.S., Soltis D., Mabberley D., Sennikov A., Soltis P.S., Stevens P.F. (2016). An update of the Angiosperm Phylogeny Group classification for the orders and families of flowering plants: APG IV. APG IV. Bot. J. Linn. Soc..

[99] Moore M.J., Hassan N., Gitzendanner M.A., Bruenn R.A., Croley M., Vandeventer A., Horn J.W., Dhingra A., Brockington S.F., Latvis M. (2011). Phylogenetic analysis of the plastid inverted repeat for 244 species: Insights into deeper-level angiosperm relationships from a long, slowly evolving sequence region. Int. J. Plant Sci..

[100] Ohba H., Bartholomew B.M., Turland N.J., Kunjun F., Kun-Tsun F. (2000). New combinations in *Phedimus* (crassulaceae). Novon.

[101] Tkach N., Röser M., Miehe G., Muellner-Riehl A.N., Ebersbach J., Favre A., Hoffmann M.H. (2015). Molecular phylogenetics, morphology and a revised classification of the complex genus *Saxifraga* (Saxifragaceae). Taxon.

[102] Xiang Q. (1995). Molecular Systematics and Biogeography of *Cornus* L. and Putative Relatives. Ph.D. Thesis.

[103] Soltis D.E., Soltis P.S., Collier T.G., Edgerton M.L. (1991). Chloroplast DNA variation within and among genera of the *Heuchera* group (Saxifragaceae): Evidence for chloroplast transfer and paraphyly. Am. J. Bot..

[104] Wen J. (1998). Evolution of the eastern Asian and eastern North American disjunct pattern: Insights from phylogenetic studies. Korean J. Plant Taxon..

[105] Soltis D.E., Kuzoff R.K. (1995). Discordance between nuclear and chloroplast phylogenies in the *Heuchera* group (Saxifragaceae). Evolution.

[106] Kawabe A., Nukii H., Furihata H. (2018). Exploring the history of chloroplast capture in *Arabis* using whole chloroplast genome sequencing. Int. J. Mol. Sci..

[107] Healey A., Lee D.J., Furtado A., Henry R.J. (2018). Evidence of inter-sectional chloroplast capture in *Corymbia* among sections Torellianae and Maculatae. Aust. J. Bot..

[108] Ogishima M., Horie S., Kimura T., Yamashiro T., Dohzono I., Kawaguchi L., Nagano A.J., Maki M. (2019). Frequent chloroplast capture among *Isodon* (Lamiaceae) species in Japan revealed by phylogenies based on variation in chloroplast and nuclear DNA. Plant Spec. Biol..

[109] Hughes M., Peng C.-I., Lin C.-W., Rubite R.R., Blanc P., Chung K.-F. (2018). Chloroplast and nuclear DNA exchanges among *Begonia* sect. *Baryandra* species (Begoniaceae) from Palawan Island, Philippines, and descriptions of five new species. PLoS ONE.

[110] Olsson S., Grivet D., Vian J.C. (2018). Species-diagnostic markers in the genus *Pinus*: Evaluation of the chloroplast regions matK and ycf1. For. Syst..

