# Supplementary material for: Next-Generation Genome Sequencing of Sedum plumbizincicola Sheds Light on the Structural Evolution of Plastid rRNA Operon and Phylogenetic Implications within Saxifragales
Source: Plants (Basel). 2019 Sep 29;8(10):386. doi: 10.3390/plants8100386 (PMC6843225; doi:10.3390/plants8100386)
Supplement: Supplementary file 1 [file plants-08-00386-s001.zip › plants-592576 supplementary/Table S1.docx]

Table 1. Genomic characteristics of 44 complete Saxifragales plastomes.

| Family | Species | Size of plastome (bp) | Size of LSC (bp) | Size of SSC (bp) | Size of IR (bp) | Total GC% | Number of genes | Number of PCGs | tRNA genes | rRNA genes | Number of rpl2 intron |
| --- | --- | --- | --- | --- | --- | --- | --- | --- | --- | --- | --- |
| Altingiaceae | *L. formosana* | 160410 | 88945 | 18917 | 26274 | 37.95 | 132 | 86 | 37 | 8 | 1 |
| Cercidiphyllaceae | *C. japonicum* | 159854 | 88013 | 18965 | 26427 | 37.92 | 129 | 84 | 36 | 8 | 1 |
| Crassulaceae | *P. kamtschaticus* | 151652 | 83000 | 16688 | 25982 | 37.76 | 132 | 87 | 36 | 8 | 1 |
| Crassulaceae | *P. takesimensis* | 147048 | 81415 | 14075 | 25779 | 38.08 | 130 | 84 | 36 | 8 | 1 |
| Crassulaceae | *R. rosea* | 151348 | 82716 | 17048 | 25792 | 37.69 | 133 | 85 | 36 | 8 | 1 |
| Crassulaceae | *S. oryzifolium* | 149609 | 79291 | 18096 | 26111 | 37.67 | 126 | 85 | 33 | 8 | 1 |
| **Crassulaceae** | ***S. plumbizincicola*** | **149397** | **81598** | **16669** | **25565** | **37.71** | **133** | **85** | **36** | **8** | **1** |
| Crassulaceae | *S. sarmentosum* | 150448 | 82212 | 16670 | 25783 | 37.75 | 132 | 86 | 36 | 8 | 1 |
| Daphniphyllaceae | *D. oldhamii* | 160137 | 88075 | 18970 | 26546 | 37.89 | 133 | 88 | 37 | 8 | 1 |
| Grossulariaceae | *R. fasciculatum* | 157298 | 86810 | 18468 | 26010 | 38.14 | 133 | 86 | 37 | 8 | 1 |
| Haloragaceae | *M. spicatum* | 158860 | 88420 | 18814 | 25813 | 36.49 | 128 | 82 | 37 | 8 | 1 |
| Hamamelidaceae | *C. bucklandioides* | 159814 | 88827 | 18179 | 26404 | 38.06 | 134 | 84 | 40 | 8 | 1 |
| Hamamelidaceae | *C. coreana* | 159398 | 87719 | 18692 | 26270 | 38.02 | 131 | 86 | 37 | 8 | 1 |
| Hamamelidaceae | *F. sinensis* | 159441 | 88124 | 18781 | 26268 | 38.13 | 133 | 87 | 37 | 8 | 1 |
| Hamamelidaceae | *H. mollis* | 159731 | 88301 | 18762 | 26334 | 37.97 | 133 | 87 | 37 | 8 | 1 |
| Hamamelidaceae | *L. subcordatum*^a^ | 158706 | 88216 | 18494 | 25998 | 38.02 | 133 | 85 | 37 | 8 | 1 |
| Hamamelidaceae | *P. subaequalis* | 159280 | 87927 | 18931 | 26211 | 37.98 | 133 | 87 | 37 | 8 | 1 |
| Hamamelidaceae | *S. henryi* | 158741 | 87507 | 18768 | 26233 | 38.17 | 136 | 85 | 40 | 8 | 1 |
| Paeoniaceae | *P. brownii* | 152228 | 84261 | 16679 | 25644 | 38.55 | 131 | 83 | 37 | 8 | 1 |
| Paeoniaceae | *P. decomposita* | 152601 | 84269 | 17036 | 25646 | 38.38 | 133 | 84 | 40 | 8 | 1 |
| Paeoniaceae | *P. delavayi* | 154405 | 82993 | 17050 | 27181 | 38.38 | 137 | 85 | 40 | 8 | 1 |
| Paeoniaceae | *P. jishanensis* | 152628 | 84292 | 17044 | 25646 | 38.36 | 132 | 85 | 37 | 8 | 1 |
| Paeoniaceae | *P. lactiflora* | 152747 | 84413 | 17034 | 25650 | 38.44 | 132 | 84 | 37 | 8 | 1 |
| Paeoniaceae | *P. ludlowii* | 152687 | 84426 | 16983 | 25639 | 38.44 | 139 | 87 | 40 | 8 | 1 |
| Paeoniaceae | *P. obovata* | 152698 | 84387 | 17027 | 25642 | 38.44 | 131 | 83 | 37 | 8 | 1 |
| Paeoniaceae | *P. ostii* | 152153 | 85373 | 17054 | 24863 | 38.32 | 123 | 84 | 31 | 8 | 1 |
| Paeoniaceae | *P. rockii* | 152821 | 84477 | 17048 | 25648 | 38.32 | 131 | 83 | 37 | 8 | 1 |
| Paeoniaceae | *P. suffruticosa* | 153119 | 84570 | 17059 | 25745 | 38.38 | 131 | 83 | 37 | 8 | 1 |
| Paeoniaceae | *P. veitchii* | 152682 | 84398 | 16978 | 25653 | 38.42 | 132 | 84 | 37 | 8 | 1 |
| Penthoraceae | *P. chinense* | 156686 | 86735 | 18378 | 25785 | 37.28 | 129 | 82 | 37 | 8 | 1 |
| Iteaceae | *I. chinensis* | 160258 | 88714 | 18252 | 26646 | 37.08 | 133 | 88 | 37 | 8 | 1 |
| Saxifragaceae | *B. scopulosa* | 156041 | 86428 | 18354 | 25585 | 37.77 | 132 | 85 | 37 | 8 | 0 |
| Saxifragaceae | *C. aureobracteatum* | 153102 | 83752 | 17316 | 26017 | 37.35 | 131 | 85 | 37 | 8 | 0 |
| Saxifragaceae | *H. parviflora* | 154696 | 85295 | 18049 | 25638 | 37.83 | 133 | 85 | 36 | 8 | 0 |
| Saxifragaceae | *H. richardsonii* | 155330 | 86060 | 18004 | 25633 | 37.79 | 130 | 83 | 37 | 8 | 0 |
| Saxifragaceae | *H. villosa* | 155361 | 86058 | 18037 | 25633 | 37.79 | 130 | 83 | 37 | 8 | 0 |
| Saxifragaceae | *M. diphylla* | 155445 | 86242 | 17945 | 25629 | 37.81 | 130 | 83 | 37 | 8 | 0 |
| Saxifragaceae | *M. formosana* | 154407 | 85506 | 17685 | 25608 | 37.95 | 130 | 83 | 37 | 8 | 0 |
| Saxifragaceae | *M. rossii* | 156960 | 87480 | 18308 | 25586 | 37.71 | 131 | 85 | 37 | 8 | 0 |
| Saxifragaceae | *O. rupifraga* | 156773 | 87391 | 18222 | 25580 | 37.75 | 131 | 85 | 37 | 8 | 0 |
| Saxifragaceae | *S. stolonifera* | 151066 | 82738 | 17504 | 25412 | 37.83 | 131 | 85 | 37 | 8 | 0 |
| Saxifragaceae | *T. cordifolia* | 155368 | 86056 | 18046 | 25633 | 37.77 | 130 | 83 | 37 | 8 | 0 |
| Saxifragaceae | *T. polyphylla* | 154902 | 86088 | 18022 | 25396 | 37.75 | 130 | 83 | 37 | 8 | 0 |
| Saxifragaceae | *T. trifoliata* | 155390 | 86149 | 17997 | 25622 | 37.78 | 130 | 83 | 37 | 8 | 0 |
