# Supplementary material for: Next-Generation Genome Sequencing of Sedum plumbizincicola Sheds Light on the Structural Evolution of Plastid rRNA Operon and Phylogenetic Implications within Saxifragales
Source: Plants (Basel). 2019 Sep 29;8(10):386. doi: 10.3390/plants8100386 (PMC6843225; doi:10.3390/plants8100386)
Supplement: Supplementary file 1 [file plants-08-00386-s001.zip › plants-592576 supplementary/Table S2.docx]

Table S2. A comparison of sizes, G + C contents in the different regions of ribosomal RNA operon from Saxifragales plastomes.

| Family | Species | 16S |  | IGa |  | trnI |  | trnI intron |  | IGb |  | trnA |  | trnA intron |  | IGc |  | 23S |  | IGd |  | 4,5S |  | IGe |  | 5S |
| --- | --- | --- | --- | --- | --- | --- | --- | --- | --- | --- | --- | --- | --- | --- | --- | --- | --- | --- | --- | --- | --- | --- | --- | --- | --- | --- |
|  |  | Size (bp)  / GC% |  | Size (bp)  / GC% |  | Size (bp)  / GC% |  | Size (bp)  / GC% |  | Size (bp)  / GC% |  | Size (bp)  / GC% |  | Size (bp)  / GC% |  | Size (bp)  / GC% |  | Size (bp)  / GC% |  | Size (bp)  / GC% |  | Size (bp)  / GC% |  | Size (bp)  / GC% |  | Size (bp)  / GC% |
| Altingiaceae | *L. formosana* | 1490 / 56.7 |  | 286 / 51.7 |  | 72 / 61.1 |  | 946 / 49.2 |  | 64 / 53.1 |  | 73 / 56.2 |  | 796 / 51.6 |  | 152 / 42.1 |  | 2811 / 55.3 |  | 98 / 59.2 |  | 103 / 50.5 |  | 248 / 44.4 |  | 121 / 51.2 |
| Cercidiphyllaceae | *C. japonicum* | 1490 / 56.8 |  | 296 / 50.3 |  | 72 / 61.1 |  | 941 / 49.0 |  | 64 / 53.1 |  | 73 / 56.2 |  | 842 / 51.4 |  | 152 / 42.1 |  | 2811 / 55.3 |  | 98 / 58.2 |  | 103 / 50.5 |  | 256 / 44.5 |  | 121 / 51.2 |
| Crassulaceae | *P. kamtschaticus* | 1490 / 56.6 |  | 296 / 50.7 |  | 73 / 60.3 |  | 942 / 49.3 |  | 68 / 48.5 |  | 73 / 56.2 |  | 837 / 50.8 |  | 152 / 41.4 |  | 2810 / 55.1 |  | 98 / 58.2 |  | 103 / 50.5 |  | 222 / 45.0 |  | 121 / 51.2 |
| Crassulaceae | *P. takesimensis* | 1490 / 56.6 |  | 296 / 50.7 |  | 73 / 60.3 |  | 942 / 49.3 |  | 68 / 48.5 |  | 73 / 56.2 |  | 799 / 51.1 |  | 152 / 41.4 |  | 2812 / 55.2 |  | 98 / 58.2 |  | 103 / 50.5 |  | 222 / 45.0 |  | 121 / 51.2 |
| Crassulaceae | *R. rosea* | 1490 / 56.5 |  | 296 / 50.7 |  | 73 / 60.3 |  | 943 / 49.1 |  | 68 / 48.5 |  | 73 / 56.2 |  | 837 / 51.0 |  | 152 / 42.1 |  | 2810 / 55.2 |  | 98 / 59.2 |  | 103 / 50.5 |  | 219 / 45.2 |  | 121 / 51.2 |
| Crassulaceae | *S. oryzifolium* | 1490 / 56.6 |  | 296 / 49.7 |  | 73 / 60.3 |  | 946 / 48.8 |  | 68 / 50.0 |  | 73 / 56.2 |  | 795 / 51.6 |  | 152 / 43.4 |  | 2809 / 55.1 |  | 98 / 60.2 |  | 103 / 50.5 |  | 219 / 44.3 |  | 121 / 51.2 |
| **Crassulaceae** | ***S. plumbizincicola*** | **1490 / 56.7** |  | **296 / 49.7** |  | **73 / 60.3** |  | **946 / 48.9** |  | **68 / 48.5** |  | **73 / 56.2** |  | **834 / 51.2** |  | **152 / 43.4** |  | **2809 / 55.1** |  | **98 / 60.2** |  | **103 / 50.5** |  | **219 / 44.7** |  | **121 / 51.2** |
| Crassulaceae | *S. sarmentosum* | 1490 / 56.6 |  | 291 / 50.5 |  | 73 / 60.3 |  | 949 / 48.7 |  | 68 / 48.5 |  | 73 / 56.2 |  | 795 / 51.2 |  | 152 / 43.4 |  | 2809 / 55.1 |  | 98 / 60.2 |  | 103 / 50.5 |  | 219 / 44.3 |  | 121 / 51.2 |
| Daphniphyllaceae | *D. oldhamii* | 1490 / 56.8 |  | 296 / 50.7 |  | 72 / 61.1 |  | 948 / 48.8 |  | 64 / 54.7 |  | 73 / 56.2 |  | 834 / 51.4 |  | 158 / 42.4 |  | 2811 / 55.2 |  | 98 / 58.2 |  | 103 / 50.5 |  | 256 / 44.1 |  | 121 / 51.2 |
| Grossulariaceae | *R. fasciculatum* | 1490 / 56.6 |  | 299 / 51.2 |  | 72 / 61.1 |  | 780 / 49.0 |  | 64 / 53.1 |  | 73 / 56.2 |  | 834 / 51.7 |  | 152 / 42.1 |  | 2811 / 55.1 |  | 98 / 58.2 |  | 103 / 50.5 |  | 264 / 43.2 |  | 121 / 51.2 |
| Haloragaceae | *M. spicatum* | 1490 / 56.6 |  | 295 / 51.2 |  | 72 / 61.1 |  | 937 / 48.3 |  | 64 / 53.1 |  | 73 / 56.2 |  | 792 / 50.8 |  | 161 / 39.8 |  | 2815 / 55.4 |  | 98 / 57.1 |  | 103 / 48.5 |  | 266 / 42.1 |  | 121 / 52.1 |
| Hamamelidaceae | *C. bucklandioides* | 1490 / 56.8 |  | 296 / 50.7 |  | 72 / 61.1 |  | 950 / 48.9 |  | 64 / 53.1 |  | 73 / 56.2 |  | 834 / 51.6 |  | 152 / 42.1 |  | 2811 / 55.3 |  | 98 / 58.2 |  | 103 / 50.5 |  | 223 / 46.2 |  | 121 / 51.2 |
| Hamamelidaceae | *C. coreana* | 1490 / 56.8 |  | 296 / 51.4 |  | 72 / 61.1 |  | 939 / 49.3 |  | 64 / 53.1 |  | 73 / 56.2 |  | 842 / 51.3 |  | 152 / 42.1 |  | 2811 / 55.0 |  | 98 / 58.2 |  | 103 / 50.5 |  | 223 / 46.6 |  | 121 / 51.2 |
| Hamamelidaceae | *F. sinensis* | 1490 / 56.9 |  | 288 / 51.7 |  | 72 / 61.1 |  | 940 / 49.4 |  | 64 / 53.1 |  | 73 / 56.2 |  | 834 / 51.6 |  | 152 / 42.1 |  | 2811 / 55.1 |  | 98 / 58.2 |  | 103 / 50.5 |  | 223 / 47.1 |  | 121 / 51.2 |
| Hamamelidaceae | *H. mollis* | 1490 / 56.8 |  | 296 / 51.0 |  | 72 / 61.1 |  | 941 / 49.3 |  | 64 / 53.1 |  | 73 / 56.2 |  | 842 / 51.3 |  | 152 / 42.1 |  | 2810 / 55.1 |  | 99 / 58.6 |  | 103 / 50.5 |  | 223 / 47.1 |  | 121 / 51.2 |
| Hamamelidaceae | *L. subcordatum*^a^ | 1490 / 56.8 |  | 288 / 50.0 |  | 36 / 58.3 |  | 931 / 49.7 |  | 25 / 56.0 |  | 73 / 56.2 |  | 842 / 51.3 |  | 152 / 42.8 |  | 2811 / 55.0 |  | 98 / 58.2 |  | 103 / 50.5 |  | 214 / 46.3 |  | 121 / 51.2 |
| Hamamelidaceae | *P. subaequalis* | 1490 / 56.8 |  | 296 / 51.0 |  | 72 / 61.1 |  | 941 / 49.3 |  | 64 / 53.1 |  | 73 / 56.2 |  | 834 / 51.6 |  | 152 / 42.1 |  | 2811 / 55.1 |  | 99 / 58.6 |  | 103 / 50.5 |  | 215 / 47.4 |  | 121 / 51.2 |
| Hamamelidaceae | *S. henryi* | 1490 / 56.9 |  | 288 / 51.7 |  | 72 / 61.1 |  | 940 / 49.4 |  | 64 / 53.1 |  | 73 / 56.2 |  | 834 / 51.6 |  | 152 / 42.1 |  | 2811 / 55.1 |  | 98 / 58.2 |  | 103 / 50.5 |  | 223 / 47.1 |  | 121 / 51.2 |
| Paeoniaceae | *P. brownii* | 1491 / 56.7 |  | 295 / 50.8 |  | 72 / 61.1 |  | 937 / 49.5 |  | 64 / 54.7 |  | 73 / 57.5 |  | 717 / 50.9 |  | 152 / 38.8 |  | 2811 / 55.1 |  | 98 / 57.1 |  | 103 / 50.5 |  | 239 / 44.4 |  | 121 / 52.1 |
| Paeoniaceae | *P. decomposita* | 1491 / 56.7 |  | 295 / 50.8 |  | 72 / 61.1 |  | 938 / 49.4 |  | 64 / 54.7 |  | 73 / 57.5 |  | 717 / 50.9 |  | 152 / 38.8 |  | 2811 / 55.1 |  | 98 / 58.2 |  | 103 / 50.5 |  | 234 / 44.9 |  | 121 / 52.1 |
| Paeoniaceae | *P. delavayi* | 1491 / 56.7 |  | 295 / 50.8 |  | 72 / 61.1 |  | 938 / 49.5 |  | 64 / 54.7 |  | 73 / 57.5 |  | 717 / 50.9 |  | 152 / 38.8 |  | 2811 / 55.1 |  | 98 / 58.2 |  | 103 / 50.5 |  | 234 / 44.9 |  | 121 / 52.1 |
| Paeoniaceae | *P. jishanensis* | 1491 / 56.7 |  | 295 / 50.8 |  | 72 / 61.1 |  | 938 / 49.4 |  | 64 / 54.7 |  | 73 / 57.5 |  | 717 / 50.9 |  | 152 / 38.8 |  | 2811 / 55.1 |  | 98 / 58.2 |  | 103 / 50.5 |  | 234 / 44.9 |  | 121 / 52.1 |
| Paeoniaceae | *P. lactiflora* | 1491 / 56.7 |  | 295 / 50.8 |  | 72 / 61.1 |  | 938 / 49.7 |  | 64 / 54.7 |  | 73 / 57.5 |  | 717 / 50.9 |  | 152 / 38.8 |  | 2811 / 55.1 |  | 98 / 58.2 |  | 103 / 50.5 |  | 234 / 45.3 |  | 121 / 52.1 |
| Paeoniaceae | *P. ludlowii* | 1491 / 56.7 |  | 295 / 50.8 |  | 72 / 61.1 |  | 938 / 49.5 |  | 64 / 54.7 |  | 73 / 57.5 |  | 717 / 50.9 |  | 152 / 38.8 |  | 2811 / 55.1 |  | 98 / 58.2 |  | 103 / 50.5 |  | 234 / 44.9 |  | 121 / 52.1 |
| Paeoniaceae | *P. obovata* | 1491 / 56.7 |  | 295 / 51.2 |  | 72 / 61.1 |  | 938 / 49.6 |  | 64 / 54.7 |  | 73 / 57.5 |  | 717 / 51.0 |  | 152 / 38.8 |  | 2811 / 55.1 |  | 98 / 59.2 |  | 103 / 50.5 |  | 234 / 45.3 |  | 121 / 52.1 |
| Paeoniaceae | *P. ostii* | 1491 / 56.7 |  | 295 / 50.8 |  | 72 / 61.1 |  | 938 / 49.4 |  | 64 / 54.7 |  | 73 / 57.5 |  | 717 / 50.9 |  | 152 / 38.8 |  | 2811 / 55.1 |  | 98 / 58.2 |  | 103 / 50.5 |  | 234 / 44.9 |  | 121 / 52.1 |
| Paeoniaceae | *P. rockii* | 1491 / 56.7 |  | 295 / 50.8 |  | 72 / 61.1 |  | 938 / 49.3 |  | 64 / 54.7 |  | 73 / 57.5 |  | 717 / 50.9 |  | 152 / 38.8 |  | 2811 / 55.1 |  | 98 / 58.2 |  | 103 / 50.5 |  | 234 / 44.9 |  | 121 / 52.1 |
| Paeoniaceae | *P. suffruticosa* | 1491 / 56.9 |  | 295 / 50.8 |  | 72 / 61.1 |  | 938 / 49.4 |  | 64 / 54.7 |  | 73 / 57.5 |  | 717 / 51.9 |  | 152 / 38.8 |  | 2857 / 55.3 |  | 98 / 57.1 |  | 103 / 51.5 |  | 234 / 44.9 |  | 121 / 52.1 |
| Paeoniaceae | *P. veitchii* | 1491 / 56.7 |  | 295 / 50.8 |  | 72 / 61.1 |  | 938 / 49.7 |  | 64 / 54.7 |  | 73 / 57.5 |  | 717 / 50.9 |  | 152 / 38.8 |  | 2811 / 55.1 |  | 98 / 58.2 |  | 103 / 50.5 |  | 234 / 45.3 |  | 121 / 52.1 |
| Penthoraceae | *P. chinense* | 1490 / 56.7 |  | 295 / 50.8 |  | 73 / 58.9 |  | 777 / 48.9 |  | 64 / 53.1 |  | 73 / 56.2 |  | 781 / 51.2 |  | 152 / 42.1 |  | 2815 / 55.2 |  | 98 / 58.2 |  | 103 / 50.5 |  | 265 / 43.0 |  | 121 / 51.2 |
| Iteaceae | *I. chinensis* | 1490 / 56.6 |  | 297 / 51.5 |  | 72 / 61.1 |  | 942 / 49.0 |  | 64 / 53.1 |  | 73 / 56.2 |  | 796 / 51.5 |  | 152 / 41.4 |  | 2811 / 55.3 |  | 98 / 58.2 |  | 103 / 50.5 |  | 224 / 44.6 |  | 121 / 51.2 |
| Saxifragaceae | *B. scopulosa* | 1490 / 56.7 |  | 296 / 51.4 |  | 72 / 61.1 |  | 948 / 48.6 |  | 64 / 53.1 |  | 73 / 56.2 |  | 834 / 51.8 |  | 158 / 41.1 |  | 2810 / 55.2 |  | 98 / 58.2 |  | 103 / 50.5 |  | 256 / 44.1 |  | 121 / 51.2 |
| Saxifragaceae | *C. aureobracteatum* | 1490 / 56.6 |  | 297 / 51.5 |  | 72 / 61.1 |  | 948 / 48.5 |  | 64 / 53.1 |  | 73 / 56.2 |  | 834 / 51.6 |  | 157 / 40.8 |  | 2810 / 55.0 |  | 98 / 59.2 |  | 103 / 49.5 |  | 216 / 46.3 |  | 121 / 51.2 |
| Saxifragaceae | *H. parviflora* | 1490 / 56.7 |  | 296 / 51.4 |  | 72 / 61.1 |  | 945 / 48.9 |  | 64 / 53.1 |  | 73 / 56.2 |  | 834 / 51.4 |  | 152 / 42.8 |  | 2810 / 55.1 |  | 98 / 59.2 |  | 103 / 50.5 |  | 256 / 44.1 |  | 121 / 51.2 |
| Saxifragaceae | *H. richardsonii* | 1490 / 56.7 |  | 296 / 51.4 |  | 72 / 61.1 |  | 945 / 48.9 |  | 64 / 53.1 |  | 73 / 56.2 |  | 834 / 51.4 |  | 152 / 42.8 |  | 2810 / 55.1 |  | 98 / 59.2 |  | 103 / 50.5 |  | 256 / 44.1 |  | 121 / 51.2 |
| Saxifragaceae | *H. villosa* | 1490 / 56.7 |  | 296 / 51.4 |  | 72 / 61.1 |  | 945 / 48.9 |  | 64 / 53.1 |  | 73 / 56.2 |  | 834 / 51.4 |  | 152 / 42.8 |  | 2810 / 55.1 |  | 98 / 59.2 |  | 103 / 50.5 |  | 256 / 44.1 |  | 121 / 51.2 |
| Saxifragaceae | *M. diphylla* | 1490 / 56.7 |  | 296 / 51.4 |  | 72 / 61.1 |  | 946 / 48.8 |  | 64 / 53.1 |  | 73 / 56.2 |  | 834 / 51.4 |  | 152 / 42.1 |  | 2810 / 55.1 |  | 98 / 59.2 |  | 103 / 50.5 |  | 224 / 45.5 |  | 121 / 51.2 |
| Saxifragaceae | *M. formosana* | 1490 / 56.7 |  | 296 / 51.4 |  | 72 / 61.1 |  | 946 / 48.8 |  | 64 / 53.1 |  | 73 / 56.2 |  | 834 / 51.3 |  | 152 / 42.8 |  | 2810 / 55.1 |  | 98 / 59.2 |  | 103 / 50.5 |  | 256 / 44.1 |  | 121 / 51.2 |
| Saxifragaceae | *M. rossii* | 1490 / 56.7 |  | 295 / 51.2 |  | 72 / 61.1 |  | 948 / 48.7 |  | 64 / 53.1 |  | 73 / 56.2 |  | 834 / 51.7 |  | 152 / 42.1 |  | 2810 / 55.2 |  | 98 / 59.2 |  | 103 / 50.5 |  | 256 / 44.1 |  | 121 / 51.2 |
| Saxifragaceae | *O. rupifraga* | 1490 / 56.6 |  | 295 / 51.2 |  | 72 / 61.1 |  | 948 / 48.7 |  | 64 / 53.1 |  | 73 / 56.2 |  | 834 / 51.6 |  | 160 / 41.3 |  | 2810 / 55.2 |  | 98 / 58.2 |  | 103 / 50.5 |  | 256 / 44.1 |  | 121 / 51.2 |
| Saxifragaceae | *S. stolonifera* | 1490 / 56.6 |  | 301 / 52.2 |  | 72 / 61.1 |  | 941 / 49.0 |  | 64 / 53.1 |  | 73 / 56.2 |  | 796 / 51.1 |  | 152 / 42.8 |  | 2810 / 55.2 |  | 98 / 57.1 |  | 103 / 50.5 |  | 260 / 44.2 |  | 121 / 51.2 |
| Saxifragaceae | *T. cordifolia* | 1490 / 56.7 |  | 296 / 51.4 |  | 72 / 61.1 |  | 945 / 48.9 |  | 64 / 53.1 |  | 73 / 56.2 |  | 834 / 51.4 |  | 152 / 42.8 |  | 2810 / 55.1 |  | 98 / 59.2 |  | 103 / 50.5 |  | 256 / 44.1 |  | 121 / 51.2 |
| Saxifragaceae | *T. polyphylla* | 1490 / 56.7 |  | 296 / 51.4 |  | 72 / 61.1 |  | 706 / 49.4 |  | 64 / 53.1 |  | 73 / 56.2 |  | 834 / 51.4 |  | 152 / 42.8 |  | 2810 / 55.1 |  | 98 / 59.2 |  | 103 / 50.5 |  | 256 / 44.1 |  | 121 / 51.2 |
| Saxifragaceae | *T. trifoliata* | 1490 / 56.7 |  | 296 / 51.4 |  | 72 / 61.1 |  | 940 / 48.9 |  | 64 / 53.1 |  | 73 / 56.2 |  | 834 / 51.4 |  | 152 / 42.8 |  | 2810 / 55.1 |  | 98 / 59.2 |  | 103 / 50.5 |  | 256 / 44.1 |  | 121 / 51.2 |

Note: ^“a”^ indicate the exon2 of trnI-GAU was absent in *L. subcordatum*.
